# 17β-Estradiol sensitizes ovarian surface epithelium to transformation by suppressing *Disabled-2* expression

**DOI:** 10.1038/s41598-017-16219-2

**Published:** 2017-12-01

**Authors:** Nhung H. Vuong, Omar Salah Salah, Barbara C. Vanderhyden

**Affiliations:** 1 Department of Cellular and Molecular Medicine, University of Ottawa, Ottawa, Canada; 2 Cancer Therapeutics Program, Ottawa Hospital Research Institute, Ottawa, Canada

## Abstract

Estrogen replacement therapy increases the risk of human ovarian cancer and exogenous estradiol accelerates the onset of ovarian cancer in mouse models. This study uses primary cultures of mouse ovarian surface epithelium (OSE) to demonstrate that one possible mechanism by which estrogen accelerates the initiation of ovarian cancer is by up-regulation of microRNA-378 via the ESR1 pathway to result in the down-regulation of a tumour suppressor called *Disabled-2* (*Dab2*). Estrogen suppression of *Dab2* was reproducible *in vivo* and across many cell types including mouse oviductal epithelium and primary cultures of human ovarian cancer cells. Suppression of *Dab2* resulted in increased proliferation, loss of contact inhibition, morphological dysplasia, and resistance to oncogene-induced senescence – all factors that can sensitize OSE to transformation. Given that *DAB2* is highly expressed in healthy human OSE and is absent in the majority of ovarian tumours, this study has taken the first steps to provide a mechanistic explanation for how estrogen therapy may play a role in the initiation of ovarian cancer.

## Introduction

Epithelial ovarian cancer (EOC) has the highest mortality rate of all cancers in the female reproductive system with a five-year survival of only 45%^1^. Women who develop the disease tend to remain asymptomatic until later stages of metastasis, but if EOC is detected early, the five-year survival rate increases to 92%^2,3^. This highlights the need to understand the initiating events of EOC so that better strategies for early detection and disease prevention can be developed. Meta-analysis of 52 epidemiological studies investigating menopausal estrogen use and EOC risk found that 55% of women who developed EOC had also used estrogen therapy^4^. In the tgCAG-TAg mouse model of EOC, 17β-estradiol (E2) was confirmed to accelerate the rate of tumour onset^5^. To follow up on these findings, this study seeks to provide a mechanistic explanation for how prolonged and consistent estrogen exposure can sensitize normal epithelial cells to transformation.

EOC is divided into multiple subtypes with epithelial EOC making up 90% of cases^6^. Many studies have shown, by investigating tumour histology, molecular profiles, and mouse models of EOC, that inclusion cysts derived from the ovarian surface epithelium (OSE) and the fimbrial fallopian tube epithelium (FTE) can be cells of origin for epithelial EOC^7^. Recent advances in high through-put techniques have allowed proteomics and genome-wide association studies to further support that both cell types are capable of giving rise to EOC^8,9^.

The OSE layer is normally a quiescent monolayer of simple squamous to cuboidal cells that surround the ovary, but they are repeatedly exposed to high levels of E2 and play an active role in ovulatory wound repair^10,11^. Little is known about the mechanisms by which E2 affects the OSE cells, but *in vivo*, it is known that OSE cells express estrogen receptors (*Esr1* and *Esr2*)^12^ and are exposed to 400-fold higher concentrations of E2 than found in serum^13^. We have previously shown that prolonged exposure to E2 can increase the incidence of morphologically dysplastic OSE where the OSE layer becomes thickened with cells having two distinct morphologies: columnar or hyperplastic. One or both of these kinds of OSE dysplasia are thought to be preneoplastic lesions because OSE expressing the oncogene SV40-T-antigen will assume a dysplastic phenotype, similar to mice treated with E2 alone, days before they invade the ovary^5^. Furthermore, E2 accelerates tumour onset in these mice, reducing the length of survival by more than 50%.

Like all epithelial cells, OSE proliferation and morphology are tightly regulated by the asymmetrical distribution of polarity proteins that provide positional cues for surface localization and growth inhibition^14^. The mechanism by which E2 causes OSE dysplasia is unknown, but we predict that E2 action is mediated, at least in part, by inhibiting a tumour suppressor gene called *Disabled-2* (*Dab2*). DAB2 is an adaptor protein that is critical for the polarized distribution of cell surface proteins (eg. CDH1)^15^, it regulates cellular response to growth control factors^16^, and it is highly expressed in normal human OSE but is lost in the majority of EOC^17,18^. Immunohistochemical staining of DAB2 in prophylactic oophorectomy tissues consistently shows a transition of DAB2+ to DAB2- cells existing on the same ovary as the OSE layer transitions from a morphologically normal monolayer to morphologically dysplastic regions. These dysplasias are associated with preneoplastic features such as multicell-layered (papillary) epithelia, invaginations, and inclusion cysts^19^.

*Dab2*(−/−) knockout mice are embryonic lethal due to extensive disorganization of embryonic cells^20^. *Dab2*(+/−) are viable and only adult female mice display aberrant cellular morphology, which is limited to uterine hyperplasia and OSE dysplasia (oviduct morphology was not reported)^21^. Given the phenotypic similarities between ovaries of *Dab2*(+/−) mice and E2-treated mice, and the loss of DAB2 in preneoplastic human ovarian tissue, we hypothesized that E2 down-regulates *Dab2* and prolonged exposure leads to epithelial disorganization and increased proliferation resulting in OSE dysplasia that could render them more susceptible to transformation. To elucidate the molecular mechanism by which E2 may sensitize normal cells to transformation, this study used primary cultures of mouse OSE cells as a model system. After the initial focus on OSE, we nevertheless demonstrated that the findings are physiologically relevant for mouse FTE *in vivo* and for human EOC cells.

## Results

### Prolonged exogenous E2 exposure causes dysplasia of the OSE monolayer

**Figure 1:**
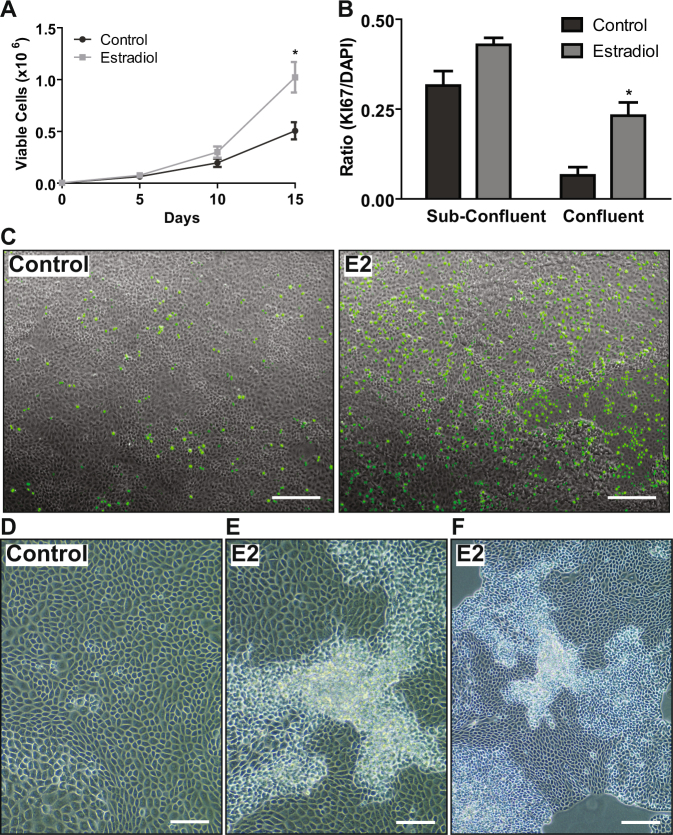
Prolonged E2 exposure causes an increase in OSE dysplasia. Primary cultures of OSE cells exposed to E2 for 15d. (**A**) Growth curve counting viable cells. (**B**) Proportion of Ki67 positive cells relative to DAPI in sub-confluent and confluent areas of the culture plate. 3–6 fields of view/group. See Supp. Fig. 5D for no primary control. (**A**,**B**) n = 3; *p < 0.05; two-way ANOVA. (**C**) Merged image of phase-contrast and IF staining of Ki67 (green) in area of confluence acquired using Zeiss Axioskop 2 microscope; scale bar = 200 µm. (**D–F**) Phase-contrast images of OSE cells on day 15 acquired using EVOS XL Core imaging system. (**D**) Confluent area in control plate; scale bar = 100 µm. (**E**) Confluent area of E2-treated cells; scale bar = 100 µm. (**F**) Sub-confluent area of E2-treated cells; scale bar = 200 µm.

Mice that received exogenous E2 via subcutaneous insertion of an E2 pellet had increased areas of hyperplastic and columnar OSE relative to mice with placebo pellets (Supp. Fig. 1), as we have reported previously^5^. This dysplastic phenotype was reproduced in tissue culture plates by maintaining OSE cells in media containing 100 nM E2 for 15d (Fig. 1). Quantification of proliferation using Ki67 staining verified that OSE cells displayed both increased number and proliferation of cells in response to E2 stimulation over 15d (Fig. 1A–C). Phase-contrast images showed that E2-treated cells formed foci of stratified cells on top of an underlying OSE monolayer after prolonged E2 exposure, whereas control cells remained as an organized monolayer (Fig. 1D–F). Foci of stratified cells were observable even in areas of sub-confluence in E2-treated dishes (Fig. 1F), demonstrating that E2-treated OSE were not becoming stratified due to over-confluence, but more likely because the mechanisms conferring proper positional cues for formation of an organized monolayer were being deregulated with prolonged E2 stimulation.

### OSE cell dysplasia is associated with a reduction in *Dab2* expression and function

**Figure 2:**
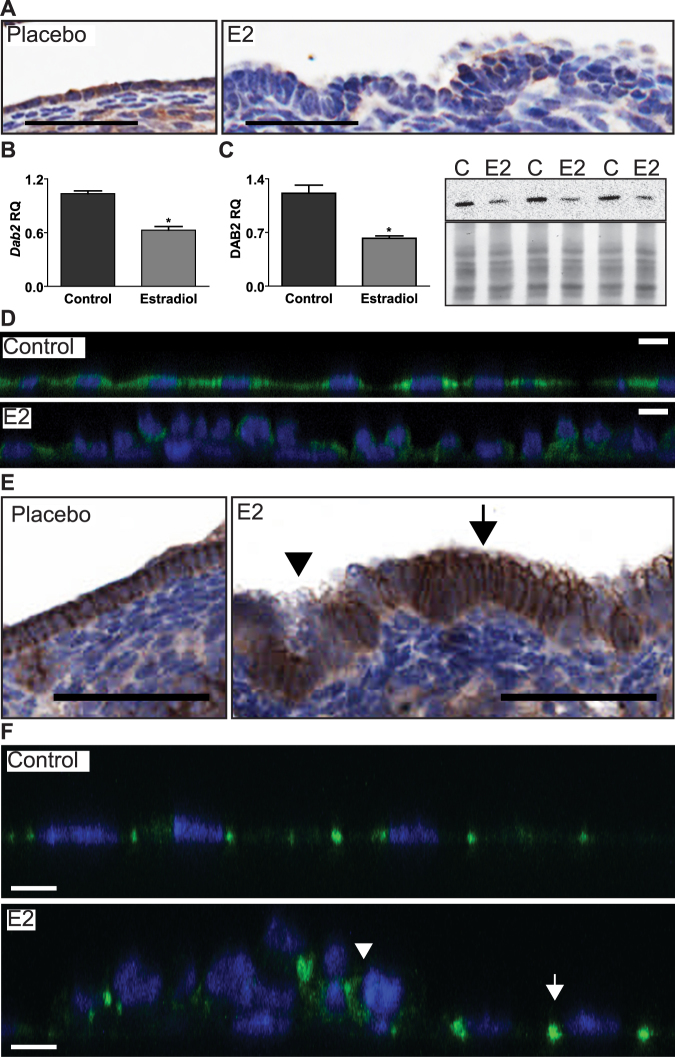
Prolonged E2 exposure suppresses *Dab2* in OSE and causes loss or mislocalization of CDH1 in stratified cells. (**A**) Mouse ovaries stained for DAB2 60d after insertion of placebo or E2 pellet. All IHC sections were stained for DAB2 in parallel on same day with identical conditions (n = 3/treatment); scale bar = 50 µm. (**B–D**) Expression of *Dab2* in OSE cells after 15d of E2 treatment in culture. (**B**) *Dab2* mRNA levels measured by qPCR. RQ = Relative Quantity. (**C**) DAB2 protein levels measured by western blot analysis. Image of blot shows results from 3 biological replicates. n = 3; *p < 0.05, paired t-test. DAB2 and Amido Black images were acquired from the same blot. (**D**) Confocal Z-stack of cultured OSE with IF staining. Blue = DAPI. Green = DAB2; scale bar = 10 µm. (**E**,**F**) Arrow heads indicate loss of expression or mislocalization of CDH1 in areas of stratified cells. Arrow indicates columnar OSE cells with increased CDH1 at lateral membrane. (**E**) Mouse ovaries 60d after insertion of placebo or E2 pellet and stained for CDH1; scale bar = 50 µm. (**F**) IF staining for CDH1 viewed by confocal microscopy of OSE after 15d of E2 treatment. Blue = DAPI; Green = CDH1; scale bar = 5 µm. (**A**,**E**) See Supp. Fig. 4 for IHC staining controls for DAB2 and CDH1. (**D**,**F**) See Supp. Fig. 5A,B for no primary controls.

OSE cells exposed to continuous E2 showed a decrease in *Dab2* expression in both animal and cell culture studies (Fig. 2A–D). IHC of ovaries from mice with E2 pellets showed loss of DAB2 associated with areas of OSE dysplasia (Fig. 2A). OSE cells exposed to E2 for 15d in culture also showed a reduction of *Dab2* at the mRNA and protein levels (Fig. 2B,C). IF staining revealed that DAB2 is globally reduced in all E2-treated cells but cells aggregated in foci of stratified cells tend to have the greatest decrease in DAB2 compared to the surrounding monolayered cells (Fig. 2D). Since DAB2 has been shown to mediate the proper localization of CDH1 in mouse endoderm^15^, mouse ovaries and OSE cells were stained for CDH1 to determine if reduced *Dab2* expression also correlates with its reduced function. It was clear that prolonged E2 exposure resulted in two distinct OSE phenotypes: (1) CDH1 expression is reduced, lost and/or mislocalized in areas of stratified cells; and (2) CDH1 expression is increased at the lateral membranes of columnar cells (Fig. 2E,F).

### *Dab2* suppression by E2 is via the ESR1 pathway and occurs before OSE dysplasia

**Figure 3:**
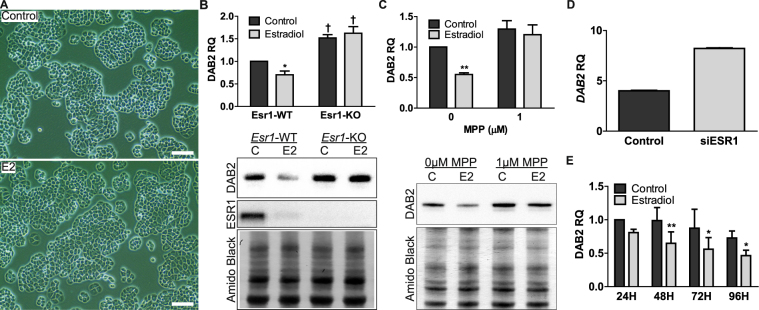
*Dab2* suppression occurs before morphological changes and is mediated through the ESR1 pathway in mouse and humans. (**A**) Phase-contrast image comparing control vs. E2-treated OSE cells at 48 h; scale bar = 100 µm. (**B**) *Esr1*-floxed OSE infected with AdGFP (*Esr1*-WT) or AdCre (*Esr1*-KO) to knockout *Esr1* were then treated with E2 for 48 h. Western blot analysis, n = 4; each replicate is from independent adenovirus infections. *E2 Significantly different relative to control; † = *Esr1*-KO significantly different relative to *Esr1*-WT; two-way-ANOVA. DAB2 and Amido Black images were acquired from the same blot. ESR1 image was acquired on separate blot using same protein lysates. See Supp. Fig. 7B for Amido Black loading control for ESR1 blot. (**C**) MASE treated with MPP (ESR1 antagonist) and 10 nM E2 for 48 h. Western blot analysis, n = 3. **p-value < 0.01, E2 significantly different relative to control; †1 μM MPP significantly different relative to 0 μM MPP; two-way ANOVA. DAB2 and Amido Black images were acquired from the same blot. (**D**) *DAB2* expression from microarray comparing MCF7 breast cancer cells (control) vs. *ESR1* silenced MCF7 cells. Data was acquired from Geoprofiles^56^. (**E**) Primary culture of human EOC ascites cells with or without treatment with E2 for up to 96 h. DAB2 expression is significantly reduced due to E2 treatment but is not changed over time. Western blot analysis, n = 3; **p-value < 0.01, *p-value < 0.05 Control vs. E2; two-way ANOVA. (**B–E**) RQ = Relative Quantity.

To determine if *Dab2* suppression is due to direct E2 stimulation or is simply a result of dysplasia, OSE cultures were treated with E2 and suppression of DAB2 protein was observed by 48 h (Fig. 3). At this time, there were no changes in cell morphology or foci of stratified cells evident (Fig. 3A). Therefore, *Dab2* suppression occurs before the E2-induced dysplasia in OSE cells.

*Esr2* transcripts were not detectable by qPCR analysis in cultured OSE cells (data not shown), suggesting that *Dab2* suppression is primarily via the ESR1 pathway. To test this hypothesis, we isolated *Esr1*-floxed OSE from mice with conditional expression of *Esr1*. *Esr1*-WT OSE readily down-regulates DAB2 following 48 h of E2 exposure but *Esr1*-KO OSE showed no change (Fig. 3B). ESR1 degradation in E2-treated cells is consistent with its known mechanism of action where ESR1 degradation by the ubiquitin–proteasome pathway is required for efficient transcription of ESR1 target genes^22^. To determine if *Dab2* suppression via the ESR1 pathway might be a more generalized mechanism, we used a different assay and cell type. MASE (mouse ovarian cancer) cells were pre-incubated with an ESR1 specific antagonist, MPP, for 1 h before treatment with E2 for 48 h. Similar to knockout of *Esr1* in OSE cells, inhibition of ESR1 in MASE cells with MPP prevented DAB2 suppression by E2 (Fig. 3C). Interestingly, both knockout of *Esr1* in OSE cells and inhibition of ESR1 by MPP in MASE resulted in a significant increase in DAB2 expression. This phenomenon was also independently observed in an ESR1-positive human breast cancer cell line where microarray data comparing control and *ESR1* siRNA transfected MCF7 cells showed that knockdown of *ESR1* significantly increased *DAB2* expression (Fig. 3D). Lastly, to further investigate if E2 regulation of *DAB2* is translatable to humans, EOC ascites cells were isolated and expanded in culture. Consistent with the results in mouse cells, DAB2 protein levels were maximally suppressed 48 h after E2 exposure (Fig. 3E).

### E2 suppresses *Dab2* by up-regulation of miR-378

**Figure 4:**
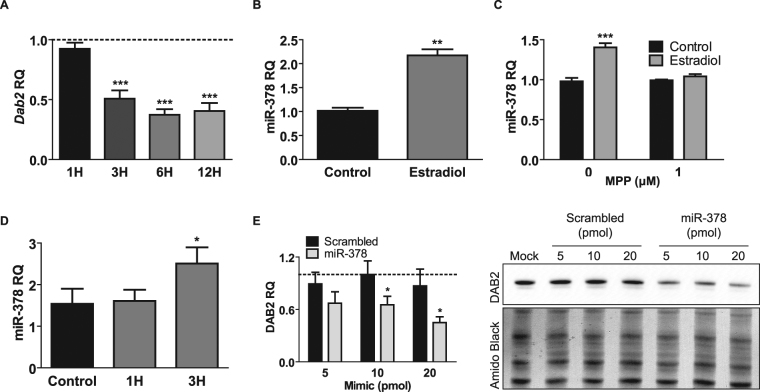
E2 suppresses *Dab2* via up-regulation of miR-378. (**A**) E2 treatment time-course with MASE cells for 1–12 h. qPCR analysis, n = 3; ***p < 0. 0.001; one-way ANOVA. Control (untreated) is normalized to 1 and is represented by dashed line. (**B**) MASE cells treated with E2 for 24 h and differential expression of miR-378 measured by qPCR analysis to validate miRNA microarray results. n = 3; **p-value < 0.01; unpaired t-test. (**C**) MASE cells treated with 10 nM E2 with/without MPP for 24 h. qPCR analysis, n = 3; ***p < 0.001; two-way ANOVA. (**D**) MASE cells treated with E2 for 1–3 h. n = 3; *p < 0.05; one-way ANOVA. (**E**) MASE cells transfected with water (Mock) or 5, 10, or 20 pmol of miR-378 mimic or mirVana miRNA Mimic Negative Control #1 (scrambled control). Western blot analysis, n = 3. *p-value < 0.05 miR-378 significantly different relative to scrambled; two-way ANOVA. Mock is normalized to 1 and is represented by dashed line. Representative blot is shown. DAB2 and Amido Black images were acquired from the same blot. (**A–E**) RQ = Relative Quantity.

Due to technical limitations of primary OSE cultures in terms of cell number yield and tolerance to transfection, MASE EOC cells were used to further investigate the mechanism by which E2 down-regulates *Dab2*. In a time-course experiment, E2 suppressed the abundance of *Dab2* transcripts to less than 50% within 3 h of E2 exposure (Fig. 4A), suggesting a direct involvement of ESR1 in *Dab2* suppression. Using chromatin immunoprecipitation, however, we found that upon E2 treatment, ESR1 did not bind to a putative estrogen response element in proximity to the transcription start site of *Dab2* that is conserved in both mouse and humans^23^ (Supp. Fig. 2). In light of these results, we investigated the possibility that *Dab2* is suppressed by microRNAs (miRNAs) that are up-regulated by E2, since recent studies have demonstrated that E2 can regulate miRNA expression^24–26^, and that miRNAs can target the *Dab2* transcript^27,28^. miRNA microarray analysis comparing control vs. E2-treated MASE cells revealed that E2 regulates the expression of 110 miRNAs (Supp. Table 1). Of the miRNAs that had the highest increase in response to E2 (>2-fold change), miR-378 was the only miRNA that had a seeding sequence capable of targeting both human and mouse *Dab2* transcripts (Supp. Fig. 3). Using qPCR analysis to validate the miRNA microarray results, we confirmed that miR-378 is up-regulated by E2 (Fig. 4B) and that this up-regulation is via the ESR1 pathway since E2 induction of miR-378 is not observed when MASE cells were pre-treated with an ESR1 antagonist (Fig. 4C). An E2 time-course experiment shows that E2 induction of miR-378 occurs within the same time frame as *Dab2* transcript suppression (Fig. 4A,D) and using miRNA mimic assays, miR-378 was confirmed to be capable of suppressing DAB2 expression (Fig. 4E).

### Loss of *Dab2* is sufficient to cause OSE cell dysplasia and prevents oncogene-associated senescence

**Figure 5:**
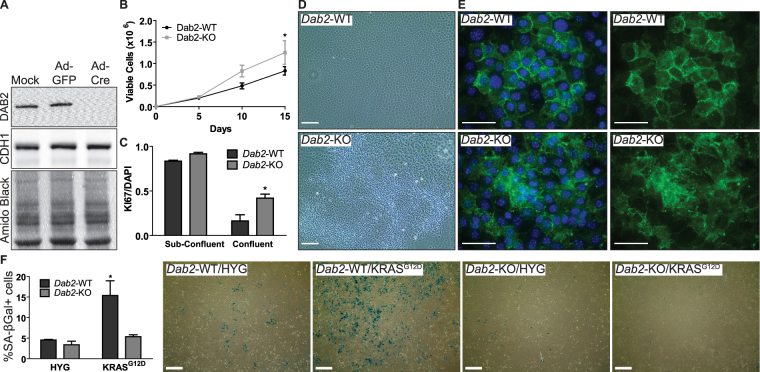
Loss of *Dab2* is sufficient to cause disorganization of OSE monolayer, increase OSE proliferation, and prevent oncogene-induced senescence. (**A**) Western blot analysis for DAB2 and CDH1 shows that *Dab2*-floxed OSE cells infected with AdGFP (*Dab2*-WT) does not affect DAB2 levels relative to mock infected cells; and that knockout of *Dab2* was successful with AdCre infection (*Dab2*-KO). Knockout of DAB2 does not change CDH1 expression. DAB2, CDH1, and Amido Black images were acquired from the same blot. (**B**) Proliferation of *Dab2*-WT and *Dab2*-KO OSE cells for up to 15d in steroid hormone-free media. n = 3; each replicate is from independent adenovirus infections. *p-value < 0.05 control vs. E2; two-way ANOVA. (**C**) Proportion of Ki67+ cells relative to DAPI in *Dab2*-floxed OSE. n = 3; *p-value < 0.05 *Dab2*-WT vs. *Dab2*-KO; two-way ANOVA. (**D**) Phase contrast images of *Dab2*-floxed OSE on day 15; scale bar = 100 µm. (**E**) IF image of *Dab2*-floxed OSE stained for DAPI (blue) and CDH1 (green); scale bar = 50 µm. See Supp. Fig. 5C for no primary control. (**F**) *Dab2*-floxed OSE infected with KRAS^G12D^ or empty vector (HYG) and stained with X-gal. Senescence-associated (SA)-βgal+ cells were identified using Image J Feulgen light green colour 2 colour deconvolution. % = SA-βgal+ pixels/total cell pixels; 3 fields of view/group (n = 3; *p-value < 0.05 *Dab2*-WT vs. *Dab2*-KO; two-way ANOVA). Representative images acquired using EVOS XL Core imaging system is shown. Scale bar = 500 µm.

To determine if the E2-induced OSE cell dysplasia is a direct result of *Dab2* suppression, primary cultures of OSE cells were isolated from mice with conditional expression of *Dab2* (*Dab2*-floxed OSE) (Fig. 5A). Results from proliferation experiments comparing *Dab2*-WT vs. *Dab2*-KO OSE cells demonstrated that even when they are cultured in steroid-free media, deletion of *Dab2* is sufficient to increase proliferation (Fig. 5B,C) and disorganization of the OSE monolayer (Fig. 5D). Ki67 staining showed that *Dab2*-WT and *Dab2*-KO OSE have similar proportions of proliferating cells when the cells are sub-confluent. However, when the cells become confluent, less than 20% of *Dab2*-WT cells are Ki67+ whereas 40% of *Dab2*-KO OSE cells remain Ki67+ despite obvious over-crowding of cells (Fig. 5C,D). Knocking out *Dab2* does not change CDH1 protein expression levels but when stained for CDH1 by IF, *Dab2*-WT cells show membrane localization of CDH1, while membrane localization is less frequently observed in *Dab2*-KO cells (Fig. 5A,E).

Oncogene induced senescence (OIS) is a phenomenon observed in culture where untransformed cells will undergo senescence in response to increased oncogenic activity in an attempt to prevent the cell’s progression into cancer^29^. To determine if loss of *Dab2* would allow OSE to escape senescence and thereby sensitize them to transformation, *Dab2*-floxed OSE were infected with a KRAS^G12D^ over-expression vector and assessed for senescence using X-gal stain. Consistent with the known ability of KRAS^G12D^ to cause OIS, *Dab2*-WT OSE expressing KRAS^G12D^ showed a significant increase in senescence-associated β-galactosidase (SA-βgal) activity while the percentage of senescent cells did not increase in *Dab2*-KO OSE (Fig. 5F).

### *Dab2* suppression by E2 is observed in mouse FTE

**Figure 6:**
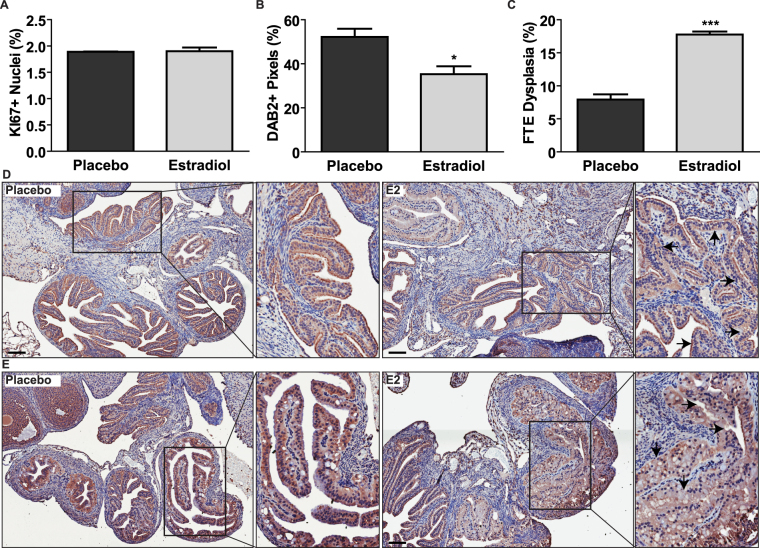
Prolonged E2 exposure results in reduced DAB2 expression and increased dysplasia of mouse FTE. (**A**) Quantification of Ki67+ nuclei over total nuclei in mouse FTE. n = 3. (**B**) Quantification of DAB2 expression in all segments of mouse FTE by pixel count using the Aperio positive pixel count algorithm (version 10.1). % = DAB2+ pixels/total pixels. n = 3; *p-value < 0.05, unpaired t-test. (**C**) Quantification of area of dysplastic FTE/total FTE area in all segments of mouse FTE using the Aperio inclusion and exclusion pen tools. n = 3; ***p-value < 0.001, unpaired t-test. (**D**,**E**) Mouse oviducts stained for DAB2 60d after insertion of placebo or E2 pellet; scale bars = 100 µm. Arrows point to areas of dysplasia. Comparison of DAB2 staining in fimbria (**D**) and ampulla (**E**) between placebo and E2-treated mice. See Supp. Fig. 4A for DAB2 IHC staining controls.

Prolonged estrogen usage has been shown to significantly increase the relative risk for epithelial EOC^4^, and with DAB2 loss observed in 85% of all EOC^17,18^, this suggests that prolonged E2 exposure and *Dab2* suppression may be relevant factors contributing to sensitizing the FTE to transformation as well. When mice were exposed to E2 for 60d, there were no changes in proliferation observed by Ki67 staining compared to placebo mice (Fig. 6A), but all segments of the FTE showed a significant decrease in DAB2 (Fig. 6B,D,E) and increased regions of dysplasia where we observed a significantly higher proportion of stratified FTE cells in the fimbria (Fig. 6D) and hypertrophy of the FTE layer in the ampulla and isthmus (Fig. 6E). It is possible for areas of stratified FTE cells to be observed as an artifact of histological sectioning due to the folding nature of the oviduct but blinded analysis of our tissue sections using the Aperio software confirms that E2-treated mice display a significantly higher proportion of dysplastic FTE relative to placebo mice (Fig. 6C). Similar to OSE cells, these results demonstrate that prolonged E2 exposure can lead to FTE dysplasia that is associated with DAB2 suppression.

## Discussion

In this study, we have demonstrated that primary cultures of OSE cells can replicate the E2-induced dysplasia observed *in vivo* and that OSE dysplasia occurs as a direct result of prolonged E2 exposure and not because of indirect E2 effects possibly occurring *in vivo*, such as altered gonadotropin secretion in the hypothalamic–pituitary–gonadal axis^5^. E2-induced OSE and FTE dysplasia can be linked to E2 suppression of *Dab2* as loss of DAB2 alone results in increased cell proliferation, disorganization (including mislocalization of CDH1), and sensitization to transformation by escaping OIS. The ESR1 pathway mediates *Dab2* suppression via E2 induction of miR-378, where *Dab2* transcripts were significantly decreased within 3 h and maximal reduction in DAB2 protein was observed after 48 h. Finally, we have demonstrated that E2 suppression of *Dab2* occurs both *in vivo* and *in vitro*, and across many cell types including mouse FTE and human ovarian and breast cancer cells. Moreover, it appears that *Esr1* can regulate *Dab2* in a ligand independent manner because in steroid hormone-free conditions, MPP inhibition of ESR1 or knockout of *Esr1* alone was sufficient to increase DAB2 expression.

miRNA microarray analysis of E2 treated MASE has identified 110 miRNA candidates whose expression is altered by E2. Since our goal was to determine if miRNA up-regulation by E2 is a possible mechanism by which *Dab2* can be decreased, we initially only focused on the top hit candidate miR-378, but it is nevertheless possible that *Dab2* suppression is a combined effort among multiple miRNAs. Using KEGG pathway analysis, we discovered that the E2-regulated miRNAs in MASE are significantly associated with synthesis and metabolism of fatty acids, the Hippo signalling pathway (the main regulator of contact inhibition), and adherens junctions (Supp. Table 2). This is not surprising as E2 is known to play major roles in fatty acid metabolism and establishing the epithelial phenotype^30,31^. This is the first time miR-378 is reported to be E2 inducible via the ESR1 pathway and a regulator of *Dab2*. Of the miRNAs that were increased >2-fold in our miRNA microarray, only miR-193b had been previously reported to be E2 regulated (in MCF7 cells)^26^. E2 regulation of miRNAs has been studied in multiple E2 responsive tissue types but little is known about E2 regulated miRNAs in ovarian epithelial cells.

Contact inhibition is a behaviour exhibited by epithelial cells to ensure proper formation of an epithelial monolayer by inhibiting cell proliferation upon contact with neighbouring cells. Loss of contact inhibition can result in outgrowth of cells from the epithelial monolayer^32^, much like the foci of stratified cells observed in our OSE model system. To determine if contact inhibition was disrupted by prolonged E2 exposure and *Dab2* loss, sub-confluent vs. confluent OSE were stained with Ki67. While *Dab2*-WT and *Dab2*-KO OSE cells showed equal proportions of proliferating cells when sub-confluent, there was a notable difference in confluent cultures where *Dab2*-KO had a higher proportion of proliferating cells relative to *Dab2*-WT OSE. The results suggest that loss of DAB2 does not alter the rate of cell proliferation per se, but DAB2 appears to play an important role in mediating contact inhibition because loss of DAB2 causes a consequent failure to form a quiescent monolayer. Loss of *Dab2* also had no apparent impact on CDH1 protein levels, but was responsible for its mislocalization. Mislocalization of CDH1 due to loss of *Dab2* is likely a contributing factor to OSE dysplasia because cell adhesion mediated by CDH1 has been shown to be an initiator for contact inhibition^33^. It is important to note that loss of DAB2 did not completely mitigate the ability of OSE cells to be contact inhibited, because *Dab2*-KO OSE still show a reduction in Ki67 positive cells as they reach confluence - albeit not to the same extent as *Dab2*-WT OSE. This is not surprising because contact inhibition is mediated by the Hippo signalling pathway receiving input from multiple sources such as adhesion molecules (i.e.: *Cdh1*, *Ctnna1*, *Ctnnb1*) and growth factors (i.e.: EGFR)^32,34^. KEGG pathway analysis of the MASE cell miRNA microarray data shows that E2 may be capable of contributing to Hippo signalling and adherens junction via miRNAs. To date, *Dab2* has only been shown to regulate CDH1 and CTNNB1 localization^15,35^.

A novel tumour suppressive role for *Dab2* was discovered using *Dab2*-floxed OSE where loss of *Dab2* allowed KRAS^G12D^ over-expressing OSE cells to bypass OIS. KRAS^G12D^-induced senescence in untransformed cells is known to be due to activation of *p53* ^36^, so it may be that DAB2 is able to impair KRAS^G12D^-induced p53 activation. *Dab2* is speculated to have a genetic interaction with *p53* where crosses between *Dab2*(+/−) and *p53*(−/−) mice resulted in sex biased embryonic lethality in females, but the mechanism by which this occurs has not been further investigated^21^.

Consistent with the phenotype of *Dab2*(+/−) mice, wherein the haploinsufficiency causes OSE dysplasia^21^, a 50% reduction in *Dab2* expression by E2 treatment was sufficient to cause OSE cell dysplasia *in vitro*. This indicates that complete loss of DAB2 is not required for OSE dysplasia to occur. *In vivo*, this change in cell morphology required prolonged suppression of *Dab2*, as OSE dysplasia was not consistently observed in conditional *Dab2*(−/−) mice until they were 6 months old^37^. In contrast, mice treated with E2 pellets showed OSE dysplasia by 60d, perhaps due to additional effects of E2 that are independent of DAB2.

The role of DAB2 in normal ovarian function is unclear, but in addition to OSE dysplasia, conditional *Dab2*(−/−) female mice have significantly reduced reproductive capacity^37^. This is likely associated with the uterine dysplasias that develop in these mice, and indicates that DAB2 plays an important role in maintaining epithelial stability in many parts of the female reproductive tract. We speculate its regulation by E2 may be a regular phenomenon during the estrous cycle. Since OSE cell proliferation has been documented to occur only before ovulation^38,39^, when E2 levels are highest, we hypothesize that transient *Dab2* suppression during estrous would allow OSE cells to increase their proliferative capacity, despite the presence of neighbouring cells. Once E2 levels drop following ovulation, re-expression of *Dab2* may play a role in re-establishing a quiescent OSE monolayer.

Little is known about the role of DAB2 in normal fallopian tube biology because previous studies investigating *Dab2*-KO mice were focused only on the ovary^21,37,40^. In humans, only one study has investigated *DAB2* expression in non-malignant FTE cells but the authors acknowledged that their study may not reflect normal fallopian tube biology because their samples were from *BRCA* mutation carriers which may have altered steroid receptor activity^41^. Here, we demonstrated that prolonged E2 exposure reduced DAB2 expression and increased areas of FTE dysplasia – similar to the OSE layer. The E2-induced FTE dysplasia was comprised of two distinct phenotypes: increased areas of stratified FTE in the infundibulum and thickening of the FTE in the ampulla and isthmus regions. In the infundibulum, there is a high ratio of ciliated to secretory FTE cells and this ratio decreases as you move away from the ovary towards the uterus, such that secretory cells predominantly line the isthmus^42^. Although both secretory and ciliated cells express ESR1, the signalling pathways regulated by E2 in these cells remain unclear^43^. Our data shows that E2 treatment does not change the proportion of Ki67+ FTE cells in E2-treated mice relative to control, consistent with previous studies showing FTE cells do not proliferate in response to E2 or ovulation^44^. E2 however, is thought to play a role in regulating the structure and function of FTE cells, where it promotes the differentiation of ciliated cells and secretory cell activity^45,46^. Studies observing porcine FTE in estrous conditions demonstrated that when E2 levels were high, FTE cells have increased height and demonstrate loss of cellular polarity relative to diestrous conditions where E2 levels are low^46^. Given that these two phenotypes are observable after 2.5d of high E2 conditions, it is not surprising that we observed increased FTE cell stratification and hypertrophy after exposing mice to E2 over 60d. Given DAB2’s known function in regulating cell polarity, we would predict that the observed reduction of DAB2 in FTE cells is contributing to their dysplastic changes.

The dysplastic changes associated with loss of DAB2, including loss of contact inhibition, increased proliferation, and escape from OIS are likely to render cells more susceptible to transformation^17–19,21,29^. This hypothesis is supported in human tissue where healthy normal OSE are DAB2+ and mostly Ki67-. As the OSE layer becomes dysplastic and progresses into cancer, the OSE becomes DAB2- and Ki67+^17,21^.

In post-menopausal women, estrogen therapy may be considered a double edged sword, for it can promote disease in the context of cancer but it is also important for maintaining women’s health to reduce the incidence of diabetes, cardiovascular disease, and osteoporosis^47–49^. This study has taken the first steps to elucidate how prolonged estrogen exposure can sensitize normal healthy OSE and FTE to transformation to offer a mechanistic explanation for the epidemiologic evidence linking estrogen therapy to increased EOC risk. In the future, these model systems can be used to identify more genes associated with early epithelial dysplasia as potential targets to investigate for disease prevention and to mitigate the risk associated with hormone therapy.

## Materials and Methods

### Mice and E2 pellet implantation

Experiments involving mice were all performed according to the Canadian Council on Animal Care Guidelines for the Care and Use of Animals on a protocol approved by the University of Ottawa Animal Care Committee. E2-pellet implant and tissue collection were performed as previously described^5^. The 0.25 mg pellet results in a serum concentration of 2.75 nM E2. A combination of FVB/N and FVB/N × C57BL/6 mice were used in this study (placebo pellet, n = 3; E2 pellet, n = 3).

### Immunohistochemical (IHC) staining of tissue

IHC was performed using a previously described protocol^50^. See Supp. Table 3 for details on antibodies used for IHC. Images were acquired using ScanScope CS2 (Leica Biosystems, Concord, Canada) and IHC quantifications were performed using the Aperio software (Leica Biosystems).

### Cells in culture

OSE cells were isolated from mouse ovaries and maintained in culture as previously described^51^. *Esr1*-floxed OSE were acquired from ovaries of *Esr1* conditional knockout mice^52^ and *Dab2*-floxed OSE were isolated from ovaries of *Dab2* conditional knockout mice^37^. *Dab2*-floxed/KRAS^G12D^ OSE were generated by infecting *Dab2*-floxed OSE with a Moloney based retroviral expression vector encoding KRAS^G12D^ and hygromycin resistance. KRAS^G12D^ expressing cells were selected using 2μg/mL hygromycin B (Sigma, Oakville, Canada). MASE EOC cells were isolated from ascites fluid of E2-treated tgCAG-TAg mice^5^. Human ascites cells (between passages 5–10) were acquired from the Ottawa Ovarian Cancer Tissue Bank from patients who consented to donate samples for research purposes.

### Adenovirus infection of *Esr1* and *Dab2* conditional knockout OSE

Conditional knockout OSE cells were suspended in serum-free αMEM medium and infected with AdGFP or AdCre (Baylor College, Houston, TX, USA) at 100pfu/cell. Infected cells were seeded into tissue culture plates and incubated at 37 °C for 1 h before FBS (10%) was added into the culture medium. Cells continued to incubate at 37 °C for 6 h, then fluid was replaced with fresh OSE medium^51^ with 5% FBS. The next day, the media were replaced and the cells were left to recover from adenovirus infection for 1 week before use in experiments.

### E2 treatment of cells in culture

Cells were seeded into tissue culture plates such that cells would be 80–90% confluent by the end of the experiment. Cells were allowed to attach overnight in OSE media. The next day, cells were washed with PBS and the media was changed to steroid-free media consisting of 5% charcoal stripped FBS in phenol-red free DMEM-F12 (Sigma), 2ng/mL EGF (Sigma), and 0.01 mg/mL ITSS supplement (Roche, Indianapolis, IN, USA). Cells were allowed to normalize to steroid-free media for 48 h before treatment with 100 nM E2 or an equivalent volume of 100% EtOH (vehicle control). Media were refreshed every 3–4 d for 15 d experiments.

### Assessment of cell proliferation

OSE cells were counted with a Vi-CELL Cell Counter (Beckman Coulter, Mississauga, Canada) 5, 10, and 15d after E2 treatment. Phase contrast images of cells during the experiment were acquired using an EVOS XL Core imaging system (Life Technologies, Burlington, Canada).

### ESR1 inhibition

1 h prior to addition of 10 nM E2, MASE cells were pre-incubated with 1 μM methyl-piperidino-pyrazole (MPP) (Sigma), or an equivalent volume of DMSO (vehicle). Cells were lysed for protein isolation 48 h after E2 stimulation.

### microRNA (miRNA) mimic assays

Lipofectamine RNAiMax (Invitrogen, Carlsbad, CA, USA) reagent was used according to manufacturer’s instructions to deliver 5, 10, or 20pmols of mirVana Mimics (ThermoFisher Scientific, Rockford, IL, USA) (Supp. Table 4) into MASE cells. Following transfection, cells were incubated for 2d at 37 °C before they were lysed for protein collection.

### Western blot analysis

Cells were lysed using M-PER (ThermoFisher Scientific) containing 1x protease inhibitor cocktail (Sigma). Protein samples were separated using NuPAGE Bis-Tris gels (ThermoFisher Scientific) and electro-transferred onto a PVDF membrane. Blots were blocked and probed as per standard protocol^53^ and visualized using ECL Substrate (Bio-Rad, Mississauga, Canada) in an chemiluminescence AlphaImager imaging system (Protein Simple, San Jose, CA, USA). “Show Saturation” tool in the AlphaImager software was used to ensure blots used for analysis or figures were not over-exposed. See Supp. Table 3 for details on antibodies used for western blot. Amido Black stain (BDH Chemicals, Poole, England) was validated for use as western blot loading control^54,55^. Image J software version 1.51o (NIH, Bethesda, MD, USA) was used for densitometric analysis of protein bands. Images generated for figures were processed in Adobe Photoshop where entire image was equally auto-contrasted then cropped to highlight bands of interest. Full-length blots are made available in Supp. Figs 6–9.

### Quantitative RT-PCR (qPCR) analysis

For mRNA analysis, mRNA was isolated from cells using an Illustra RNA extraction kit (GE Healthcare, Ottawa, Canada) and reverse-transcribed using an iScript cDNA Synthesis Kit (Bio-Rad). iTaq Universal Probes Supermix (Bio-Rad) with PrimeTime Probes (IDT, Coralville, IA, USA) or SsoFast EvaGreen Supermix (Bio-Rad) with primers (Invitrogen) was used to run qPCR analysis on the 7500Fast system (Applied Biosystems, Foster city, CA, USA). For miRNA analysis, miRNA was isolated from cells using a miRNeasy miRNA isolation kit (Qiagen, Toronto, Canada) and TaqMan MicroRNA assays (Applied Biosystems) were performed according to manufacturer’s protocol to run qPCRs using the 7500Fast system. See Supp. Table 4 for miRNA assays and primers used for qPCR.

### Immunofluorescence (IF) staining

Cells were seeded onto glass coverslips and treated with E2 for up to 15d as described above. Cells were fixed, permeabilized, blocked, and probed according to antibody datasheet instructions, then mounted onto slides using ProLong Gold mountant with DAPI (ThermoFisher Scientific). See Supp. Table 3 for details on antibodies used for IF. Wide-field IF images were acquired using a Zeiss Axioskop 2 microscope and confocal images were acquired using a Zeiss LSM 510 confocal microscope system (Zeiss, Toronto, Canada). Image J software was used to quantify Ki67 staining using the cell counter plugin.

### miRNA microarray

Data and details for miRNA microarray of MASE cells treated with E2 for 24 h (n = 3) can be found on Geoprofiles: http://www.ncbi.nlm.nih.gov/geo/query/acc.cgi?acc=GSE98391. http://www.microrna.org was used for target prediction analysis.

### Statistical analysis

Statistical analyses were performed using GraphPad Prism 5 (GraphPad software, La Jolla, CA, USA). One-way ANOVAs had significance determined with Dunnett’s Multiple Comparison Test and two-way ANOVA had significance determined with a Bonferroni multiple comparisons post-test. All ANOVAs performed were with repeated measures test. T-tests were all two-tailed. All graphs show mean values and error bars represent the s.e.m.

### Data availability

The data that support the findings of this study are available from the corresponding author upon request.

## Electronic supplementary material

Supplemental Data
