# Supplementary material for: 17β-Estradiol sensitizes ovarian surface epithelium to transformation by suppressing Disabled-2 expression
Source: Sci Rep. 2017 Dec 1;7:16702. doi: 10.1038/s41598-017-16219-2 (PMC5711839; doi:10.1038/s41598-017-16219-2)
Supplement: Supplementary file 1 — Supplemental Data [file 41598_2017_16219_MOESM1_ESM.pdf]

Supplemental Data

17β-Estradiol sensitizes ovarian surface epithelium to transformation by suppressing *Disabled-2* expression

Nhung H. Vuong[1, 2], Omar Salah Salah[2], and Barbara C. Vanderhyden[1, 2, *]

1. Department of Cellular and Molecular Medicine, University of Ottawa
2. Cancer Therapeutics Program, Ottawa Hospital Research Institute

* Corresponding author

Corresponding author's contact information:

Dr. Barbara Vanderhyden (613 737 7700 extension 70330)

501 Smyth Road, 3rd Floor, Box 926

Ottawa, Ontario, Canada, K1H 8L6

bvanderhyden@ohri.ca

Supplemental Materials and Methods

Chromatin Immunoprecipitation (ChIP): MASE cells were treated with 100nM E2 for 45min then cross-linked with 1% formaldehyde for 30min at RT. Cross-linking was quenched by adding 125mM glycine for 10min at RT. Cells were sonicated with a Bioruptor (Diagenode, Denville, NJ, USA) for 30sec on high and 60sec off for 80 cycles at 4°C to produce 150-200bp fragments. To bind antibody to Dynabeads magnetic beads (Thermofisher Scientific, Nepean, Canada), 5µg of ESR1 anti-body (Santa Cruz, sc-542, Mississauga, Canada) or normal mouse IgG (control for non-specific interactions) (Millipore, Temecula, CA, USA) was incubated with 20µl of magnetic beads overnight at 4°C the day prior to immunoprecipitation (IP). 250µg of DNA was used per IP and DNA was pre-cleared using 1µl/ml salmon sperm (Sigma, Oakville, Canada), 10µl/ml ovalbumin (Sigma, Oakville, Canada), and 10µl/ml Dynabeads magnetic beads for 1h at 4°C. 10% of pre-cleared chromatin was saved as “Input” and IP of DNA with antibody-bound beads occurred overnight at 4°C. Next day, the beads bound by immune-complexes were collected using a Magna GrIP Rack (Millipore, Temecula, CA, USA) and washed twice with low-salt, high-salt, and LiCl washes. The IP materials were eluted from the beads by incubating the samples with elution buffer at 65°C for 10min at 1400rpm in a thermomixer (Eppendorf, Mississauga, Canada). Reverse cross linking and protease treatment was performed on each sample including input. The IP genomic DNA fragments were isolated via phenol:chloroform:isoamyl alcohol extraction, precipitated with ethanol, then pelleted. Air-dried DNA pellet was resuspended in nuclease-free water. HotStarTaq DNA Polymerase Kit (Qiagen, Toronto, Canada) in combination with primers spanning the mouse *Greb1* ERE1 and ERE2 were used to amplify DNA. Amplified PCR products were run on a 4% agarose gel containing RedSafe Nucleic Acid Staining Solution (Intron Biotechnology, Toronto, Canada) and bands were visualized with an EpiChem II Darkroom transilluminator (UVP Laboratory, Upland, CA, USA). *Dab2* ERE forward primer: GTGTTCACGCAAGAACAGACAG; *Dab2* ERE reverse primer: TGCTGACTTCAGGAGAAGGGT; *Greb1* ERE forward primer: ACCGCAAACTGTATCAGTGG; *Greb1* ERE reverse primer: CCAGCTGCCACAATTAGAAAC; *Gapdh* promoter forward primer: TACTCGCGGCTTTACGGG; *Gapdh* promoter reverse primer: TGGAACAGGGAGGAGCAGAGAGCA.

Supplemental Figures

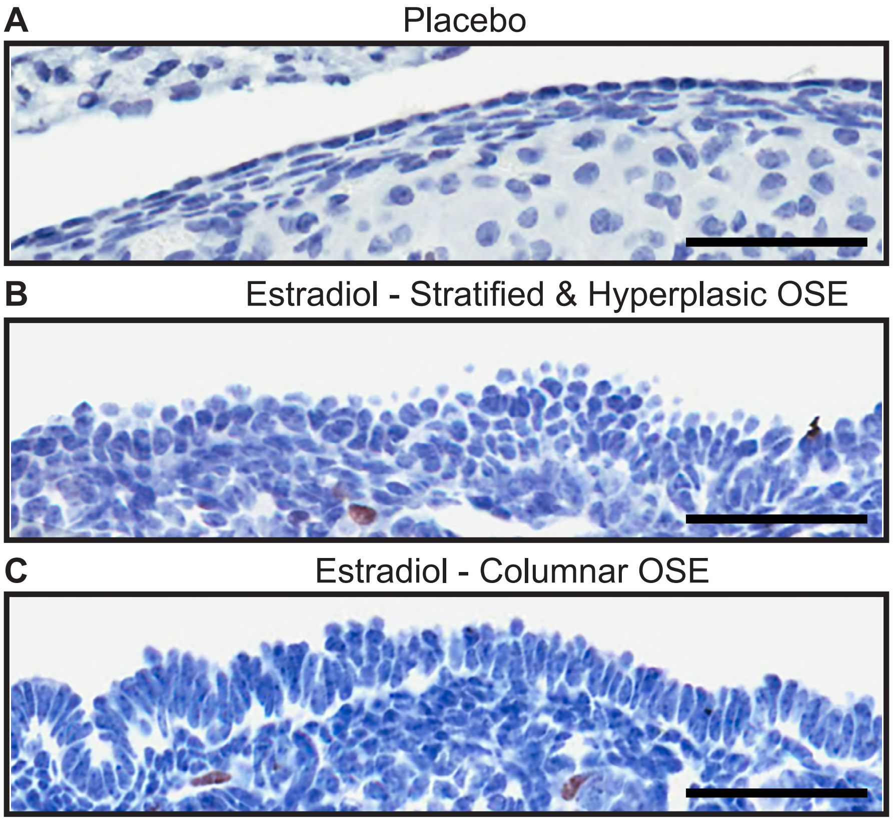

Supplemental Figure 1: Prolonged E2 stimulation alters OSE cell morphology. Representative images of OSE layer from FVB/n mice 60d after implantation of placebo **(A)** and E2 **(B-C)** pellet. **(B)** Area of stratified and hyperplasic OSE. **(C)** Area of columnar OSE. Scale bar=50µm.

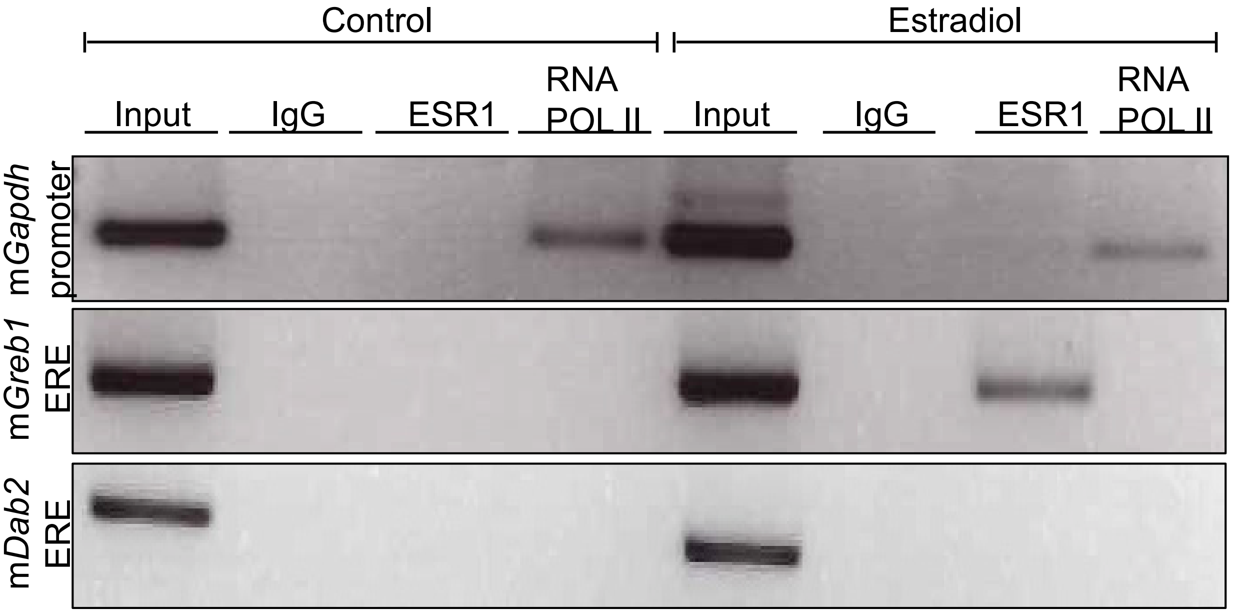

Supplemental Figure 2: The putative ERE proximal to the transcription start site of *Dab2* does not bind ESR1. ChIP with ESR1 does not show enrichment of the putative *Dab2* ERE in E2-treated MASE cells. Primers spanning the mouse *Greb1* ERE (a known ESR1 target) were used as a positive control for the ESR1 antibody. Pull-down of RNA POL II with the mouse *Gapdh* promoter (a constitutively expressed gene) confirms that the ChIP assay was successful for both treatment groups. IP with normal mouse IgG did not show enrichment of non-specific interactions.

```
      3' ggaagACUGAGGUUCAGGUCa 5'  mmu-miR-378        3' ggUGAGAAGACUGAGGUUC-AGGUCa 5'  hsa-miR-378
         | | |    |||||||                                |||||   |||    ||| |||||
1082: 5' uccagUCAUUGUUAGUCCAGg 3'  Mouse Dab2    915: 5' ccACUCUCUUGAGCUGAAGUUCCAGa 3'  Human DAB2
```

Supplemental Figure 3: Alignment of miR-378 relative to *Dab2* transcript in mouse and humans.

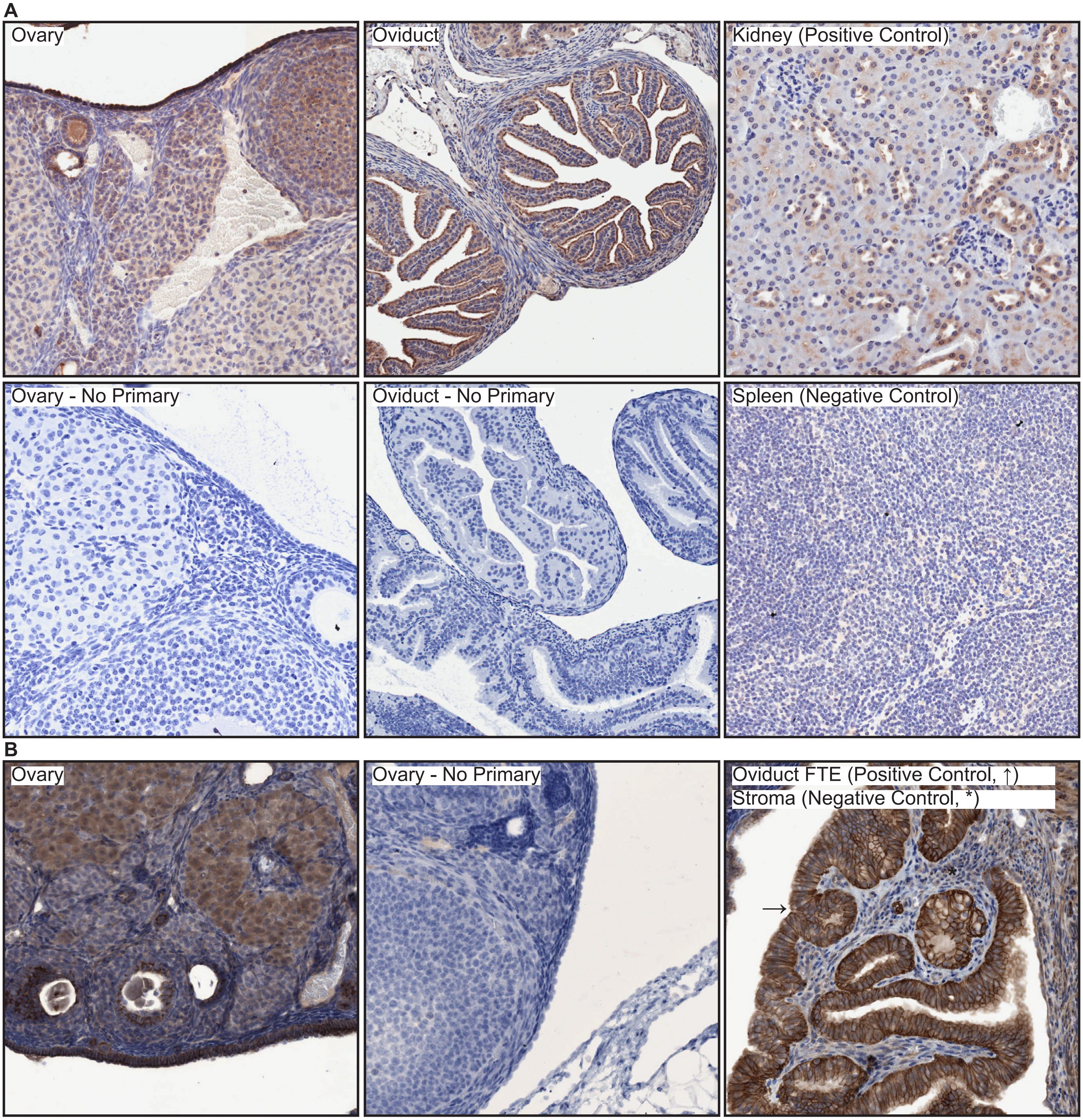

Supplemental Figure 4: IHC Controls for (A) DAB2 and (B) CDH1.

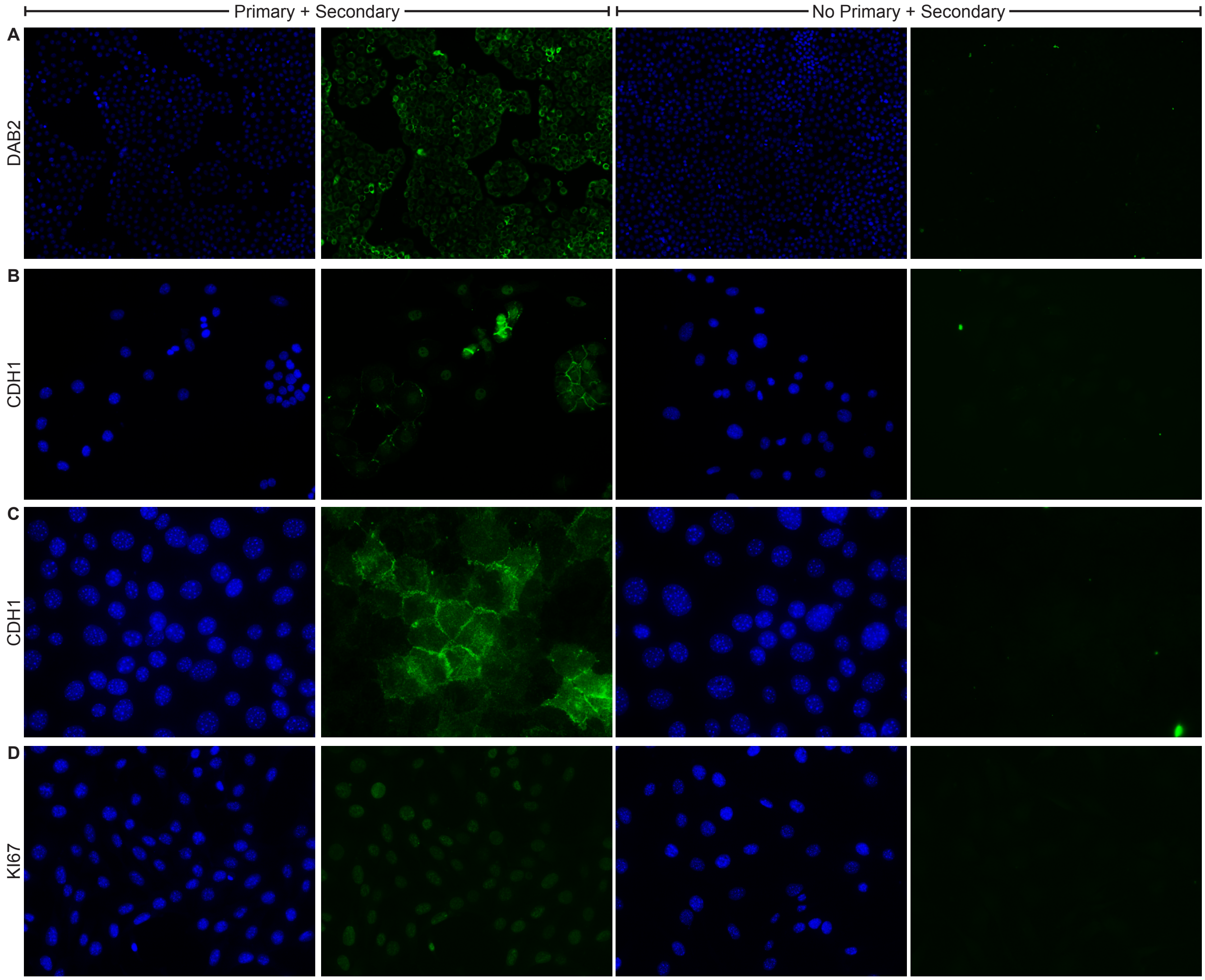

Supplemental Figure 5: OSE DAB2, CDH1, and KI67 IF staining “no primary” controls. Representative images showing the level of background observed for each antibody used for IF in this study. **(A)** Anti-DAB2. **(B)** Anti-CDH1; Abcam (Cat#53033); discontinued antibody used in Figure 2. **(C)** Anti-CDH1; Abcam (Cat#76055); Antibody used in Figure 5. **(D)** Anti-KI67.

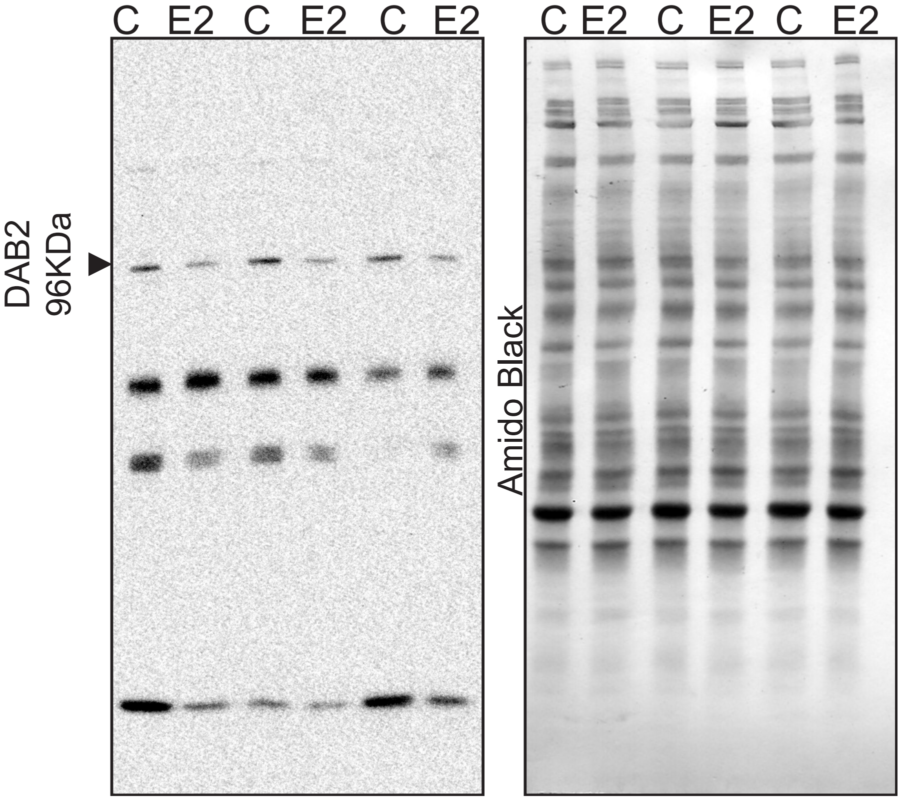

Supplemental Figure 6. Full DAB2 and Amido Black loading control western blots from Fig.2C. Additional bands are from probing blot with other antibodies.

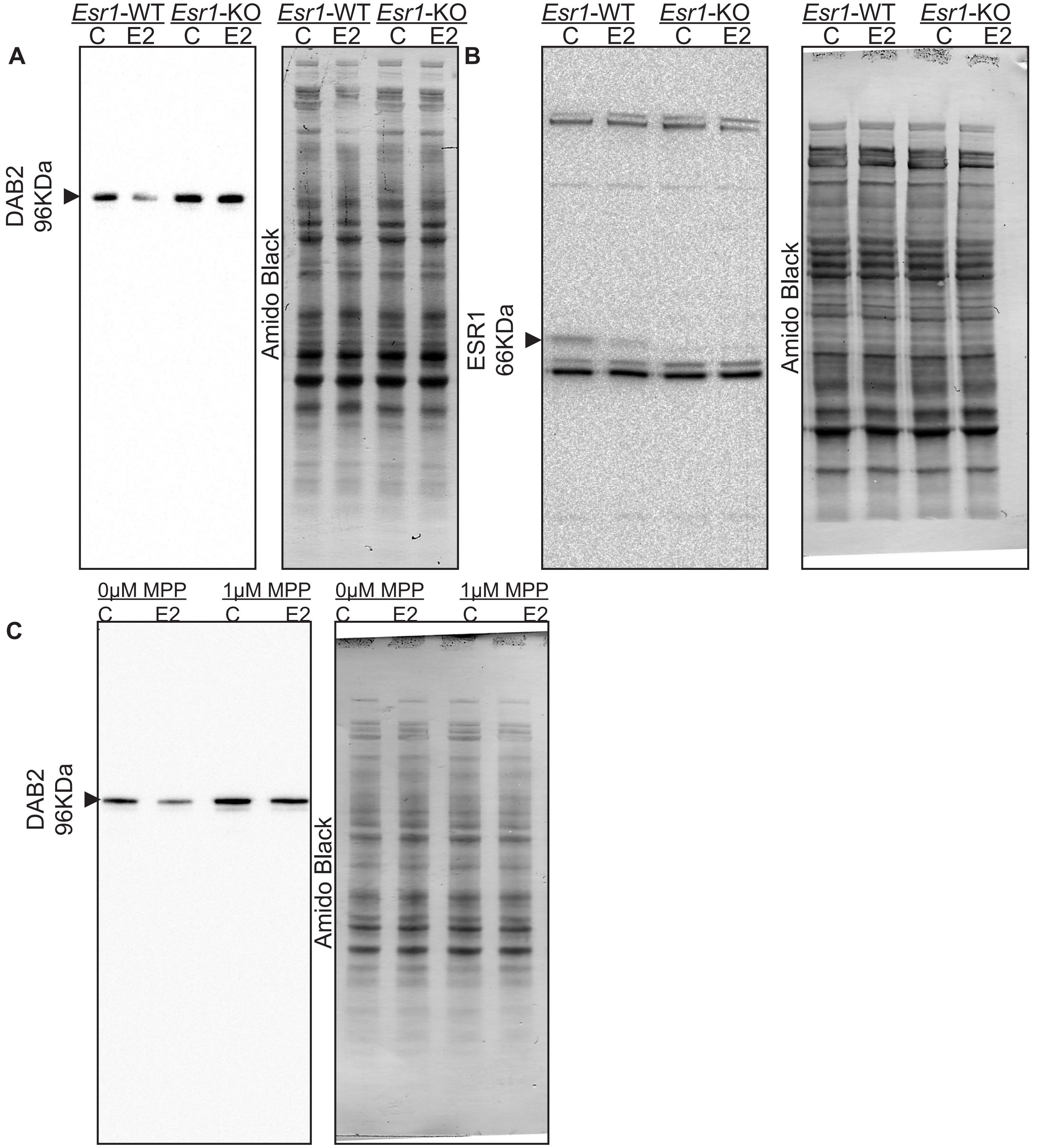

Supplemental Figure 7. Full western blots from Figure 3. **(A)** Fig. 3B DAB2 and Amido Black loading control blots. **(B)** Fig. 3B ESR1 and Amido Black loading control blots. **(C)** Fig. 3C DAB2 and Amido Black loading control blots.

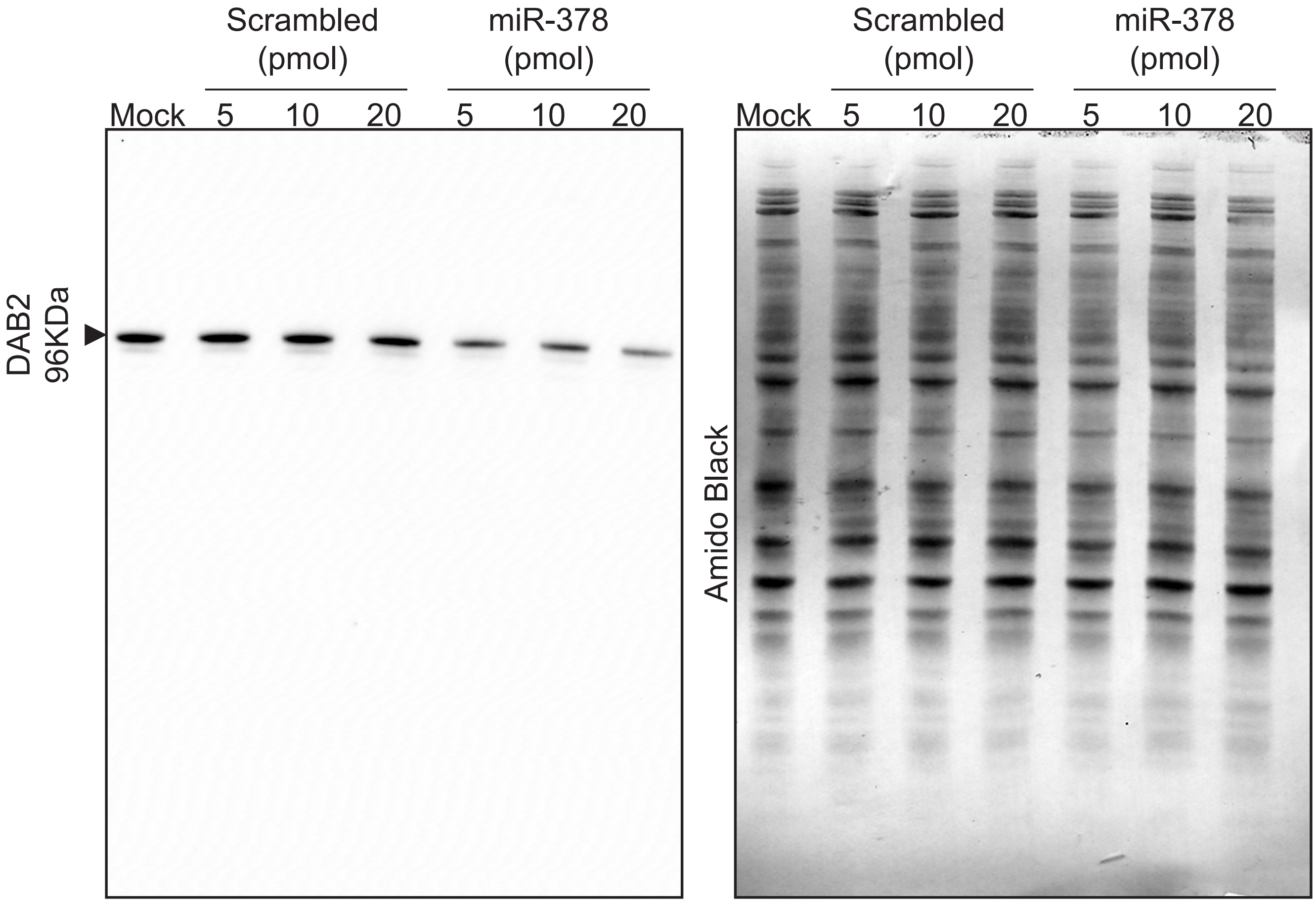

Supplemental Figure 8. Full DAB2 and Amido Black loading control western blots from Fig. 4E.

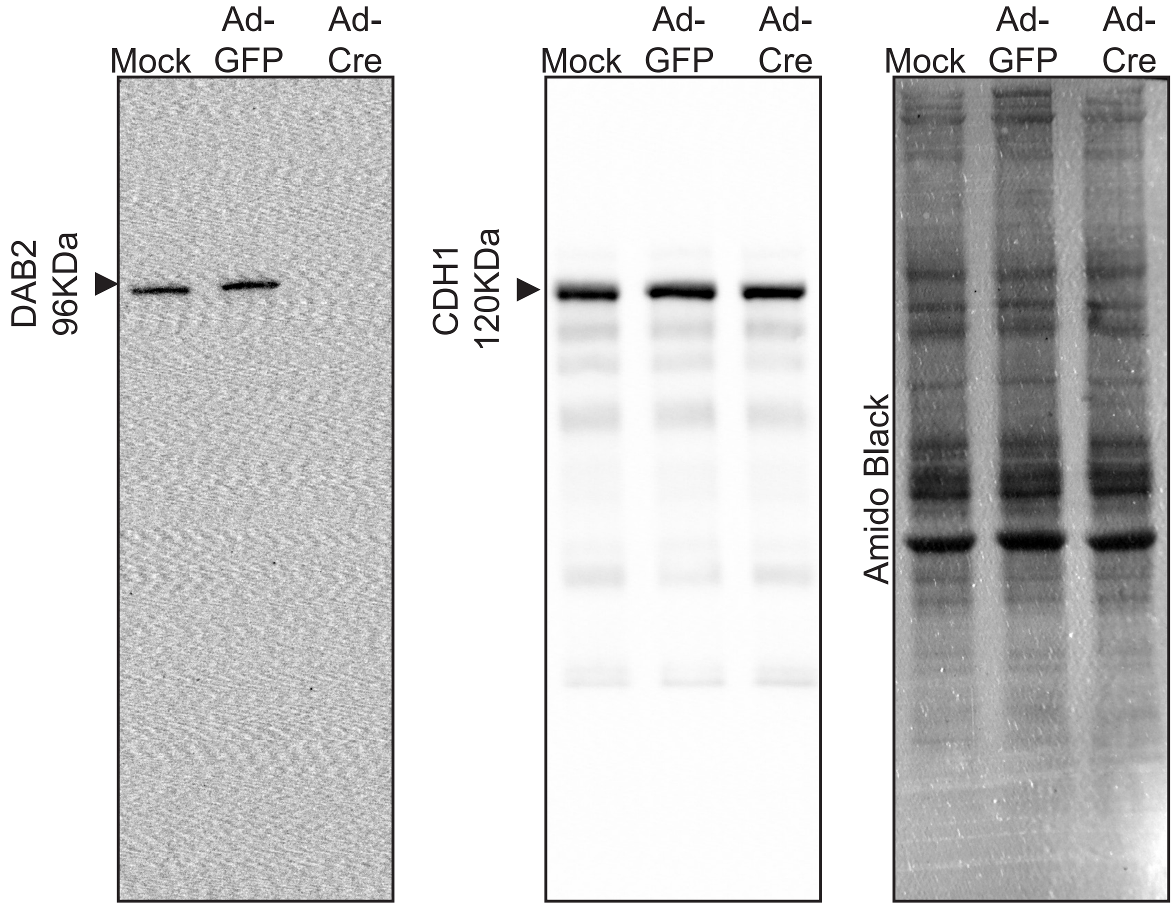

Supplemental Figure 9. Full DAB2, CDH1, and Amido Black loading control western blots from Fig. 5A.

<u>Supplemental Tables</u>

Supplemental Table 1: miRNA microarray results from MASE cells treated with or without E2 for 24h (n=3). Paired one-way repeated measure ANOVA was used and only significantly changed miRNAs are shown (p-value<0.05). Data are sorted by fold change (FC).

miRNA Name	FC	P.Value
miR-6984-5p	5.524	5.0E-02
miR-7007-5p	4.875	4.3E-02
miR-5121	4.698	4.5E-02
miR-6368	3.52	4.1E-02
miR-1894-5p	3.248	2.7E-03
miR-7082-5p	2.761	4.8E-02
miR-1231-5p	2.661	2.9E-02
miR-7016-3p	2.325	2.6E-02
miR-193b-3p	2.134	5.5E-05
miR-378c	2.072	9.9E-05
miR-378d	2.065	5.8E-05
miR-378a-3p	2.005	1.3E-04
miR-24-1-5p	1.95	2.3E-02
miR-378b	1.923	2.4E-05
miR-5622-3p	1.765	3.1E-02
miR-7115-5p	1.755	3.5E-02
miR-1907	1.732	3.7E-02
miR-466i-5p	1.701	2.3E-02
mir-5121	1.66	4.7E-03
miR-5128	1.657	3.3E-02
miR-365-2-5p	1.654	1.2E-02
miR-6942-5p	1.598	7.8E-04
miR-185-3p	1.569	2.4E-02
miR-15a-3p	1.567	3.7E-02
mir-3113	1.539	2.9E-02
miR-125b-2-3p	1.485	3.2E-02
miR-7001-5p	1.463	3.0E-02
miR-425-3p	1.461	3.6E-02
mir-7684	1.445	4.2E-02
miR-6993-5p	1.425	1.3E-02
miR-3092-5p	1.392	2.4E-02
miR-7031-5p	1.389	1.1E-02
miR-6958-5p	1.347	2.7E-02
miR-293-3p	1.326	2.7E-02
miR-133c	1.326	7.3E-03
miR-299a-5p	1.324	1.4E-02
miR-3572-3p	1.316	5.0E-02
mir-16-2	1.306	3.1E-02
miR-672-5p	1.304	4.9E-02
mir-3971	1.299	1.5E-02
miR-692	1.299	1.4E-02
mir-181d	1.295	5.0E-02
miR-707	1.294	2.2E-02

miRNA Name	FC	P.Value
miR-7646-5p	1.292	4.1E-02
mir-99a	1.282	9.7E-03
mir-6900	1.278	2.5E-02
mir-1894	1.277	4.2E-02
miR-466n-5p	1.271	5.0E-02
miR-670-5p	1.27	3.6E-02
mir-1933	1.269	4.1E-02
mir-690	1.269	1.4E-02
miR-6359	1.263	4.5E-02
miR-7234-5p	1.25	4.2E-02
miR-207	1.248	1.5E-02
miR-541-5p	1.242	3.1E-02
miR-3971	1.237	4.2E-02
mir-5623	1.236	2.4E-02
miR-147-5p	1.231	2.8E-02
mir-1952	1.23	3.2E-02
miR-7074-3p	1.229	3.3E-02
miR-7232-5p	1.217	2.4E-02
miR-6918-3p	1.217	2.3E-02
mir-7662	1.216	3.3E-02
mir-1839	1.212	4.4E-02
miR-6985-3p	1.209	4.3E-02
miR-6949-3p	1.208	4.4E-02
mir-1195	1.206	2.8E-02
mir-3069	1.204	4.0E-02
miR-3070b-3p	1.201	3.9E-02
mir-3475	1.189	4.6E-02
mir-192	1.167	4.9E-02
miR-216b-3p	1.167	4.9E-02
miR-7021-3p	0.841	3.3E-02
miR-7089-5p	0.838	5.0E-02
miR-370-5p	0.828	4.7E-02
mir-6925	0.823	2.8E-02
mir-7057	0.823	4.3E-02
miR-7220-3p	0.822	4.0E-02
mir-301a	0.818	4.9E-02
miR-489-3p	0.813	2.6E-02
miR-7233-3p	0.812	3.3E-02
miR-190b-5p	0.811	3.4E-02
mir-3084-1	0.806	2.2E-02
miR-5107-3p	0.798	3.4E-02
miR-6912-3p	0.791	1.7E-02
miR-1945	0.787	3.0E-02

miRNA Name	FC	P.Value
miR-1197-3p	0.787	1.6E-02
miR-669c-3p	0.787	3.5E-02
miR-3112-5p	0.785	1.8E-02
miR-7224-3p	0.777	3.8E-02
miR-6408	0.774	2.4E-02
miR-6962-3p	0.767	1.1E-02
miR-6481	0.766	1.3E-02
mir-7027	0.764	3.9E-02
miR-194-5p	0.758	5.0E-03
miR-6397	0.756	1.8E-02
miR-547-5p	0.755	6.6E-03
miR-3106-3p	0.752	7.7E-03
miR-466d-5p	0.743	4.4E-03
mir-212	0.741	6.9E-03
miR-7051-5p	0.729	2.8E-03
miR-322-5p	0.723	3.7E-02
miR-466p-5p	0.709	4.3E-03
miR-376c-3p	0.708	4.7E-02
miR-7079-5p	0.7	1.2E-02
miR-344d-1-5p	0.678	4.2E-02
miR-1947-3p	0.667	2.4E-02
miR-331-5p	0.653	2.9E-03
miR-6236	0.649	1.6E-02
miR-339-3p	0.536	1.9E-02

Supplemental Table 2: Top five pathways associated with E2 responsive miRNAs as determined by KEGG pathway analysis of significantly changed mature microRNAs from MASE cells treated with or without E2 for 24H. DIANA-miRPath v3.0 software and the DIANA-microT-CDS algorithm was used to perform analysis[1].

KEGG pathway	p-value	#genes	#miRNAs
Fatty acid biosynthesis	5.75E-10	7	14
Fatty acid metabolism	8.22E-10	24	36
Hippo signaling pathway	1.31E-09	90	58
Biosynthesis of unsaturated fatty acids	1.77E-08	15	28
Adherens junction	2.13E-07	49	47

Supplemental Table 3: Antibodies.

Primary Antibody						Secondary Antibody					
Application	Target	Antibody	Company	Catalog#	Dilution	Antibody	Conjugate	Company	Catalog#	Dilution	Note:
IHC	DAB2	anti-DAB2 rabbit polyclonal	Proteintech Group (Rosement, IL, USA)	10109-2-AP	1:100	DAKO EnVision+ System (rabbit)	HRP	Dako (Carpinteria, CA, USA)	K4003	N/A	
IHC	KI67	anti-KI67 rabbit monoclonal	Abcam (Toronto, Canada)	ab16667	1:200	DAKO EnVision+ System (rabbit)	HRP	Dako (Carpinteria, CA, USA)	K4003	N/A	
IHC	CDH1	anti-CDH1 mouse monoclonal	BD Biosciences (Missisauga, Canada)	610181	1:400	DAKO EnVision+ System (mouse)	HRP	Dako (Carpinteria, CA, USA)	K4000	N/A	Immunostaining for CDH1 was performed according to the mouse-on-mouse kit (Vector, Brockville, Canada)
Western Blot	DAB2	anti-DAB2 rabbit polyclonal	Proteintech Group (Rosement, IL, USA)	10109-2-AP	1:2000	Goat anti-rabbit IgG (H + L)	HRP	Bio-rad, (Missisauga, Canada)	1721019	1:5000	
Western Blot	ESR1	anti-ESR1 rabbit polyclonal	Santa Cruz Biotechnology (Missisauga, Canada)	sc-542	1:100	Goat anti-rabbit IgG (H + L)	HRP	Bio-rad, (Missisauga, Canada)	1721019	1:5000	
IF	DAB2	anti-DAB2 rabbit polyclonal	Santa Cruz Biotechnology (Missisauga, Canada)	sc-13982	1:100	Goat anti-Rabbit IgG (H+L) Superclonal Secondary	Alexa Fluor 488	Invitrogen, (Carlsbad, CA, USA)	A27034	1:1000	
IF	CDH1	anti-CDH1 rabbit polyclonal	Abcam (Toronto, Canada)	ab53033	1:400	Goat anti-Rabbit IgG (H+L) Superclonal Secondary	Alexa Fluor 488	Invitrogen, (Carlsbad, CA, USA)	A27034	1:1000	Discontinued; Used in Fig. 2
IF	CDH1	anti-CDH1 mouse monoclonal	Abcam (Toronto, Canada)	ab76055	1:100	Goat anti-Mouse IgG (H+L) Superclonal Secondary	Alexa Fluor 488	Invitrogen, (Carlsbad, CA, USA)	A28175	1:1000	Used in Fig. 5
IF	KI67	anti-KI67 rabbit monoclonal	Abcam (Toronto, Canada)	ab16667	1:100	Goat anti-Rabbit IgG (H+L) Superclonal Secondary	Alexa Fluor 488	Invitrogen, (Carlsbad, CA, USA)	A27034	1:1000	

Supplemental Table 4: Probes, primers, and miRNA assays.

Product	Target	Species	Company	Catalog #	Assay ID	Probe	Forward Primer	Reverse Primer
PrimeTime Predesigned qPCR Probes	Dab2	Mouse	Integrated DNA Technologies (Coralville, Iowa, USA)	N/A	Mm.PT.58.42543573	FAM/CTGCT-GCCA/ZEN/ TTCCCTT-GAGTTTCAT/ 3IABkFQ	GCTTGT-GTTGTCCCT-GAGAG	GTATTGATGAT-GTGCCTGATGC
	Tbp	Mouse	Integrated DNA Technologies (Coralville, Iowa, USA)	N/A	Mm.PT.39a.22214839	FAM/ACTT-GACCT/ZEN/ AAAGAC-CATTGCACTTC-GT/3IABkFQ	CCAGAACT-GAAAATCAAC-GCAG	TGTATC-TACCGT-GAATCTTGGC
Custom qPCR Primers	Dab2	Human	Invitrogen (Nepean, Canada)	N/A	N/A	N/A	TGTATC-TACCGT-GAATCTTGGC	CAAGCAAGT-CATTGGCTGAA
	TBP	Human	Invitrogen (Nepean, Canada)	N/A	N/A	N/A	GAACATCAT-GGATCAGAA-CAACA	ATAGGGATTC-CGGGAGTCAT
TaqMan MicroRNA Assay	miR-378	Mouse Human	ThermoFisher Scientific (Nepean, Canada)	4427975	002243	N/A	N/A	N/A
	snoRNA202	Mouse	ThermoFisher Scientific (Nepean, Canada)	4427975	001232	N/A	N/A	N/A
mirVana miRNA Mimic	miR-378	Mouse	ThermoFisher Scientific (Nepean, Canada)	4464066	MC12581	N/A	N/A	N/A
	Negative Control #1	Mouse Human	ThermoFisher Scientific (Nepean, Canada)	4464058	N/A	N/A	N/A	N/A

Supplemental References

1. Vlachos, I. S. *et al.* DIANA-miRPath v3.0: deciphering microRNA function with experimental support. *Nucleic Acids Res.* **43,** W460–W466 (2015).
